# Enhancing trauma education worldwide through telemedicine

**DOI:** 10.1186/1749-7922-7-S1-S4

**Published:** 2012-08-22

**Authors:** Antonio C Marttos, Fernanda M Kuchkarian, Phillipe Abreu-Reis, Bruno MT Pereira, Francisco S Collet-Silva, Gustavo P Fraga

**Affiliations:** 1 University of Miami Miller School of Medicine, Surgery Department (D40), PO Box 016960 Miami, FL 33101, USA; 2 Federal University of Parana, Rua XV de Novembro, 1299, CEP 80.060-000, Curitiba, Parana, Brazil; 3 University of Campinas (Unicamp), Faculty of Medical Sciences, Division of Trauma Surgery, (FCM/Unicamp), R. Alexander Fleming, 181, Cidade Universitaria “Prof. Zeferino Vaz”, CEP: 13.083-970, Campinas, São Paulo, Brazil; 4 Hospital das Clínicas da Faculdade de Medicina Universidade de Sao Paulo, Av. Dr. Arnaldo, 455, Cerqueira Cesar, CEP: 01.246-903, São Paulo, Brazil

## Abstract

Advances in information and communication technologies are changing the delivery of trauma care and education. Telemedicine is a tool that can be used to deliver expert trauma care and education anywhere in the world. Trauma is a rapidly-evolving field requiring access to readily available sources of information. Through videoconferencing, physicians can participate in continuing education activities such as Grand Rounds, seminars, conferences and journal clubs. Exemplary programs have shown promising outcomes of teleconferences such as enhanced learning, professional collaborations, and networking. This review introduces the concept of telemedicine for trauma education, and highlights efforts of programs that are utilizing telemedicine to unite institutions across the world.

## Introduction

Advances in telemedicine now allow trauma specialists to remotely care for patients anywhere in the world. Telemedicine is the use of telecommunications technology to provide healthcare services at a distance [1] Telehealth, a closely related term, encompasses a broader definition to include activities beyond clinical services such as education and administrative services [2]. Telemedicine provides unique opportunities to meet some of the challenges of contemporary trauma education. At the core of such technologies is videoconferencing, which is frequently used to deliver trauma care and education in real-time. In addition to meeting trauma educational needs, telemedicine is promoting international collaborations that promise to revolutionize the way trauma care is delivered on a population-based level. This paper will review the use of telemedicine in trauma, with emphasis on education. Experience implementing trauma tele-educational activities from our respective institutions will be highlighted.

## Telemedicine for trauma

In recent years, there has been tremendous growth in the field of telemedicine. Due to a combination of technology-driven market forces, as well as increasing demands for improvements in the global health sector; these advances are providing the tools necessary to enhance medical care and education. Telemedicine in trauma can be used for the routine monitoring of patients [3], to austere environments and large-scale disasters [4]. Examples of telehealth services include specialist consultations, remote patient monitoring, continuing education, and referral services. Wide adoption of telemedicine and telehealth promises increased access to quality trauma care, while simultaneously reducing costs. At its fundamental core, telemedicine is based on the ethical principle that quality care should be made available to all people, anywhere and at anytime.

The trauma, emergency and critical care fields are facing multiple challenges worldwide. Issues with overcrowding, increased demands for trauma care, lack of funding, and a lack of disaster preparedness have been identified as chief concerns [5]. Of particular concern is the continued workforce shortage, including shortage of specialists and nurses. Researchers estimate that there will be significant shortages of physicians across several surgical specialties [6]. As population increases, it is estimated that there will be a deficit of 6,000 general surgeons by 2050 [7]. Several factors have been identified as contributors to the shortage; including barriers to recruitment of medical students into general surgery residencies, and general dissatisfactions with lifestyle concerns. In trauma care there are inherent discrepancies, particularly between rural and urban areas. Inadequate access to trauma is a reality for many populations. Despite research that patients have better outcomes when treated at designated trauma centers, many hospitals around the world that provide injury care are not such facilities [8]. Providers often lack the resources and experience to treat trauma patients, due to either low volume of severely injured patients and/or limited training opportunities. In addition, rural hospitals do not have sufficient access to subspecialty care for instance orthopedics and neurosurgery. These factors can cause unintended delays in the diagnosis and treatment of trauma patients, resulting in poorer outcomes such as increased morbidity and length of stay. At these moments, the ability to have a more experienced trauma specialist available through telemedicine for a consultation is invaluable.

The advent of telemedicine use for trauma and emergency care developed out of the need to address such disparities. Telemedicine facilitates access to care for traditionally underserved populations in remote areas with fewer health services. Trauma surgeons can now remotely assist in the evaluation and care of patients. There are many studies demonstrating the clinical effectiveness of teletrauma applications in rural settings [9-11]. Perhaps the most significant effect is the decrease in time to treat trauma patients. Patients can be either treated locally with the assistance of a remote expert or quickly transferred to an appropriate center. This has significant cost-reducing potential for healthcare systems as well as patients and their families; as costly transfers can be minimized when appropriate avoiding further financial and social burdens.

## Rationale

Technology is revolutionizing how health professionals obtain information. The constantly evolving state of medicine makes efficiently obtaining information a necessity. In trauma care, teams of physicians and other clinicians frequently rely on a flow of information using a multitude of communication modes. New surgical techniques and procedures, heavy emphasis on trauma care protocols and evidence-based medicine naturally lead to the use of telemedicine to disperse new knowledge in a timely fashion. This is especially beneficial when resident education and rural providers are considered. Due to the geographical misdistribution of health professionals, rural providers often face professional isolation that can result in knowledge and skill attrition [12]. Physical distance from other specialists, regional hospitals, and continuing education programs prevent remote practitioners from staying up-to-date. Work-hour limitations and changes in training duration for residency programs have challenged educators to find innovative solutions to overcome limited faculty resources and time while also improving the quality of medical education [13].

## Telemedicine in surgical education

There are considerable applications of telemedicine for surgical education and training. At the center of such applications is the use of videoconferencing (VC). VC first was first used to broadcast a surgical procedure overseas in 1962 [14]. Since then, VC has been embraced as an effective tool among surgeons to discuss surgical procedures as well as to conduct telementoring and teleconsultations [15]. The educational process in surgery is essentially composed of training and manual abilities development supervised by a more experienced surgeon who acts as a teacher [16]. However, many surgical procedures (i.e. open abdominal/thoracic trauma surgery) are difficult for learners to visualize the maneuvers of the surgeon due to field view limitations. The introduction of laparoscopy was a milestone in the teaching of surgery mainly by allowing images shared between observers, tutors and residents in real time [17]. The use of robot-observers is a paradigm shift for open surgery teaching, in which cameras can be used for images transmission as a new tool in surgeons’ training [18].Through telemedicine, students and residents can observe the procedure from a remote classroom [15]. Studies show that students feel more comfortable to ask questions, learn more, and have fewer questions not answered by faculty [19]. Furthermore, reducing the number of people in the OR results in is less noise and distraction for the surgical team [20]. VC has also been examined for surgical follow-up care, burns, and wound management.

**Figure 1:**
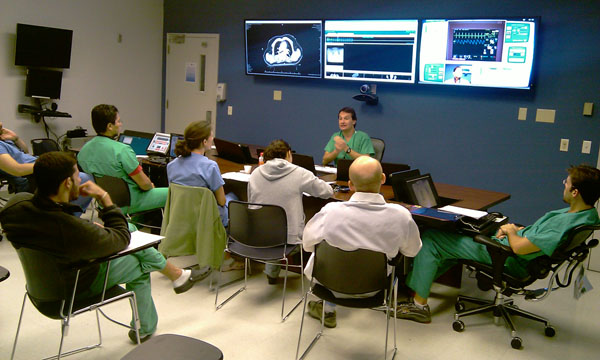
Use of telemedicine during daily rounds at University of Miami/Ryder Trauma Center in Miami.

Interactive remote support can help health staff improve the management of patients as well as enhance the educational value of daily patient care activities, such as with patient rounds. At the University of Miami/Ryder Trauma Center in Miami, FL, use of telemedicine for daily morning rounds is currently standard operating procedure in the Trauma Intensive Care Unit (TICU) [21]. In replacement of traditional bedside rounds, the TICU team uses a mobile videoconferencing telemedicine system (Figure 1). The technology used for daily rounds is the InTouch Health’s RP-7 System, a wireless mobile robotic platform that includes a remote Control Station. The Control Station software consists of a joystick that can be used to maneuver the robot remotely. Clinicians are able to remotely view the patient, look at vital signs, ventilator settings, and examine laboratory and imaging data--all from one single location. The remote location is fitted with multiple large screens and computers to display patient information to an audience of clinicians. An important outcome of tele-rounds is that it helps reduce the spread of infections associated with heavy bedside traffic, while maintaining the educational integrity of traditional rounds [22].

## Examples of current initiatives in trauma tele-education

The experiences gained through the use of VC in surgical education have paved the way to incorporate its use in other areas of trauma education. There are several initiatives to expand trauma education through telemedicine occurring at multiple international sites. Earlier initiatives consisted of using integrated services digital networks (ISDN) for data transmission modes. Through the use of ISDN videophones, a “virtual residency” was established in Canada between a rural and urban hospital [23]. Their collaborations include teleconsultations and biweekly trauma rounds to provide continuing medical education to rural providers. In Europe, six University hospitals in four countries (Switzerland, Belgium, Germany and France) held weekly surgical teleconferences and reported their experiences over a two-year period [24]. The authors measured the accuracy of telediagnosis by randomly selecting surgical cases to be reviewed by a panel of surgeons. The authors found that the real-time transmission of documents, combined with interactive discussion increased diagnostic accuracy.

In recent years, VC via ISND use has been reduced considerably due to declining equipment costs and increases in Internet protocol (IP)-based and 3G mobile phones solutions. Since then, several small to large-scale networks that link trauma centers, academic center, tertiary care hospitals and clinics have been developed. It is estimated that in the United States alone, there are currently 200 existing telemedicine networks, each with varying degrees of activity and capacity [25] Some networks are local, while others are statewide. Notable examples are seen in Florida [26], Utah [27], Arizona [28] and California [29]. Through telemedicine networks, health professionals at multiple sites can interact with one another, collaborate on projects, and attend professional meetings. Continuing education activities can occur such as Grand Rounds, case presentations and seminars. In Brazil, the telemedicine network named RUTE (University Network of Telemedicine, available from http://rute.rnp.br) has been connecting university hospitals around the country, with the objective to create a more uniform surgical medical education of these health professionals [30]. This national network supports existing telemedicine projects as well as provides incentives for inter-institutional collaborations.

**Figure 2:**
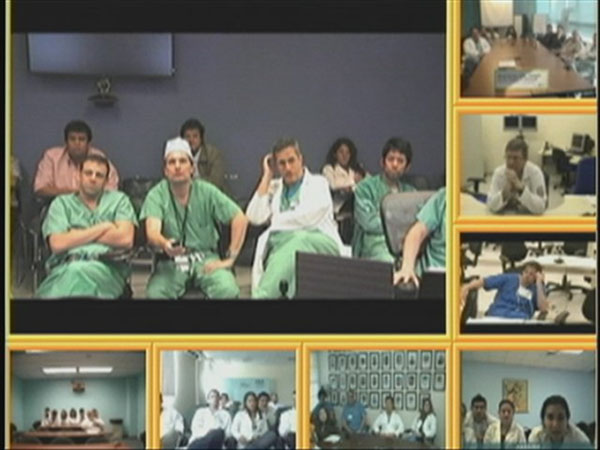
Tele-Grand Rounds organized every Friday discussing trauma cases from different institutions.

Together with several institutions around the world, the University of Miami/Ryder Trauma Center has established the International Trauma Tele-Grand Rounds. Through videoconferencing, complex trauma case presentations and advanced trauma and critical care topics are discussed on a weekly basis. Case presentations provide students, residents, fellows and attending physicians with an outstanding tool for education and sharing of medical expertise across borders. Continuing medical education (CME) credits are available to eligible physicians. To date, there have been 42 participating institutions from the United States, Brazil, Colombia, Bahamas, Haiti, Canada, Venezuela, Argentina, Panama, Puerto Rico, Dominican Republic, British Virgin Islands, Spain, Thailand, Turkey and Iraq; ranging from academic medical centers to urban trauma centers, military, community and rural hospitals. The Panamerican Trauma Society has adopted the Tele-Grand Rounds as one of their educational activities (Figure 2). The Clinical Hospital of University of Campinas (Unicamp), in Campinas, Brazil, reported their experiences participating in 100 videoconferencing meetings over a one-year period in all specialities [31]. Trauma surgery meetings accounted for the majority of the teleconferences. Through the results of the program’s success, telemedicine is now an integral part of their trauma surgical residency curriculum.

An additional innovative use of telemedicine for education is with the rise of remote “journal clubs”. With the huge number of articles published daily worldwide, it is a challenge to surgeons with a busy practice to keep themselves up-to-date. Through telemedicine, the Brazilian Society of Integrated Trauma Care (SBAIT) and the Brazilian College of Surgeons (CBC) have joined forces with the University of Toronto, Canada to promote Evidence-Based Telemedicine – Trauma and Acute Care Surgery (EBT-TACS) [32]. These are regular meetings for literature review of topics most relevant to surgeons. Participants select ahead of time a scientific article for review, and conduct in-depth analysis of the study design, outcomes, strengths and limitations. Subsequently recommendations are disseminated in the Journal of the CBC. These meetings make it possible for non-academic physicians who practice in smaller centers to stay up-to-date, as well as promote critical analysis of evidence-based surgical topics.

## Discussion

Telemedicine, as an expanding technology, is creating previously unimagined possibilities for the reality of health care providers. There is now a way to extend the reach of a trauma surgeon anywhere in the world. This extension reduces limitations imposed on distant providers as well as patients. With high-speed data linked to video units, specialists can now take care of patients in distant hospitals who normally would not have access to such services. This ability has tremendous cost-saving potential, as well as for improved patient outcomes. Patients who do not require transfer can be treated locally when a remote expert can assist the local team. In addition, if the patient does need to be transferred, the remote expert can also ensure that the patient is stable.

Telemedicine also offers a solution to address the disparities in access to trauma education. Experiences from using VC for surgical education have broadened its use to a wider scope and audience. Today VC can be used for consultations, patient rounding, mentoring and continuing medical education. Providers in rural or remote areas can have access to educational opportunities available to those in large, urban academic settings. Studies have shown that the use of telemedicine for trauma education facilitates resident training, enhances communication and enriches the educational experience. The diversity of institutional settings allows participants to learn from others on how to best treat trauma patients, despite differences in resources and expertise. In addition to serving as an educational tool, the series provides a mechanism for physicians to network and collaborate on future endeavors. All of this leads will lead to a more robust, educated workforce.

Many telehealth programs have been developing across the world. Some of them however, find difficulties in sustaining their activities once program funding ends. Adding an educational component to a telehealth program may ensure its sustainability in the long-run. The synergy created by different institutions participating in teleconferences for example, can lead to other collaborations in the future. In addition, as physicians become more accustomed to being on video, they can then be better prepared to communicate with patients in the same way.

## Conclusion

The development and advancement of telemedicine over the past years have opened doors to an immense number of possibilities. Not only has telemedicine been used for consultation, diagnosis and treatment purposes; it is also being used in distance and continuing medical education. Institutions are developing a variety of web-based distance learning programs as well as formal grand rounds and lectures using telemedicine technology. In particular, telemedicine can be used to overcome disparities in training and education and to deliver higher-quality health care to patients in remote locations. Telemedicine will not only extend the reach of the trauma education but it will also help bridge the gap between limited resources, lack of available staff and reduced budget across many specialties in medicine.

## Competing interests

The authors declare that they have no competing interests.

## Authors’ contributions

AM, GF, FC, and BP provided subject matter expertise and assistance with the literature. FK was responsible for preparing and editing the manuscript. All authors read the manuscript.
